# Aerial Jumping in the Trinidadian Guppy (*Poecilia reticulata*)

**DOI:** 10.1371/journal.pone.0061617

**Published:** 2013-04-16

**Authors:** Daphne Soares, Hilary S. Bierman

**Affiliations:** Department of Biology, University of Maryland, College Park, Maryland, United States of America; Claremont Colleges, United States of America

## Abstract

Many fishes are able to jump out of the water and launch themselves into the air. Such behavior has been connected with prey capture, migration and predator avoidance. We found that jumping behavior of the guppy *Poecilia reticulata* is not associated with any of the above. The fish jump spontaneously, without being triggered by overt sensory cues, is not migratory and does not attempt to capture aerial food items. Here, we use high speed video imaging to analyze the kinematics of the jumping behavior *P. reticulata.* Fish jump from a still position by slowly backing up while using its pectoral fins, followed by strong body trusts which lead to launching into the air several body lengths. The liftoff phase of the jump is fast and fish will continue with whole body thrusts and tail beats, even when out of the water. This behavior occurs when fish are in a group or in isolation. Geography has had substantial effects on guppy evolution, with waterfalls reducing gene flow and constraining dispersal. We suggest that jumping has evolved in guppies as a behavioral phenotype for dispersal.

## Introduction

The kinematics of swimming has been a subject of interest for biologists for many decades. Researchers have examined various aspects of underwater locomotion of fishes from the physics of fin propulsion, buoyancy and drag and thrust, to muscle physiology and the adaptation of body morphology (for a review, see [1]). Less is known about the jumping behavior of fishes. Fishes have been reported to jump out of the water for three reasons: to catch non-aquatic prey, to avoid predation from below and to negotiate obstacles in migration routes. Here, we examine the jumping kinematics of the Trinidadian guppy, *Poecilia reticulata*, and propose that the jumping observed in this species may have evolved for another reason.

### I. Jumping in Fishes

Some fish species jump to consume non-aquatic food items. This strategy allows fishes to exploit arboreal and terrestrial prey, such as insects, spiders, and a variety of small vertebrates. One such example comes from archer fishes. These fish are well known for their ability to target prey with a bolus of water [2]–[4], but they are also able to jump and catch prey in midair. This kinematics of jumping has been described for the aerial prey-capture maneuvers of the archer fish *Toxotes microlepis* [5]. That fish jumps vertically out of the water from rest to capture prey, using a short thrust production phase generated by the caudal fin followed by a drop in acceleration to a motionless glide phase achieving greater heights with greater numbers of tail strokes. Shih and Techet [5] (2010) reported that *T. microlepis* jump up to 2.5 body lengths (fishes measured 6.8 to 11.1 cm) with velocities of up to 1.4 m/sec. The fish reaches its maximum velocity in 20 milliseconds and the parabolic trajectory of the jump overshoots the prey before descending into the water.

The osteoglossid Silver Arowana (*Osteoglossum bicirrhosum*) like archer fishes, leaps from the water to ambush prey resting on low-hanging branches. Lowry et al. [6] (2005) compared the kinematics of *O. bicirrhosum* feeding under water and in the air and reported that aerial feeding events proceed more quickly (9.2 vs. 3.0 body lengths/sec) than those in the water. These authors also reported that in aerial feeding events, the fish increases its swimming speed as it approaches the prey and, when it is within approximately one body length of the prey, it bends its body into an S-shaped posture prior to striking. Fish are out of water approximately 1 body length. Other studies have mentioned aerial feeding in the four-eyed fish, *Anableps anableps* [7], the rivulus, *Rivulus hartii* [8], the Atlantic salmon, *Salmo salar*, and the sea trout, *Salmo trutta* [5], [9], but none has specifically examined the associated kinematics in detail.

Fishes also leap into the air to escape predators. The ability to escape predators is critical for individual fitness and is presumed to be under intense selective pressure [10]–[13]. At least three unrelated families of fishes have evolved aerial excursions to avoid predation. However, involvement of the brainstem startle circuitry, including the Mauthner cells, has yet to be determined for each family. Mauthner cells are a pair of large neurons that innervate the axial musculature to produce the unilateral tail-flip or C-start reflexes in teleost fish.

The marine flying fishes exhibit long glides in the air [14]–[17]. Fish probably fly mainly to escape from predators, particularly dolphins and squid, although these fish may also jump as an energy-saving strategy for cruising long distances [18]. Adult flying fish vary in size (15–50 cm body length) and are broadly divided into two categories: ‘two-wingers’ in which the enlarged pectoral fins make up most of the lifting surfaces (for example *Fodiator, Exocoetus, Parexocoetus*), and ‘four-wingers’ in which both pectoral and pelvic fins are hypertrophied, such as in *Cypsilurus* and *Hirundichthys*. Four wingers have been more thoroughly studied and these fish swim toward the water surface at high speed (<30 body lengths/sec) with the lateral fins adducted, leap through the water surface at a shallow angle, accelerate to take-off speed by taxiing with the lateral fins abducted and the tail beating in the water (∼50 beats/sec; [17]0. Flying fish do not flap their pectoral of pelvic fins to gain lift. Fish in the way can ascertain great distances. *Cypsilurus californicus* for example, a four winger that measures ∼45 cm in length, produces aerial bouts that will reach heights of up to eight meters (∼20 body lengths), traveling great distances (∼30 m; ∼60 body lengths; [17]).

The freshwater African butterfly fish, *Pantodon buchholzi* (family Pantodontidae), and the hatchet fish, *Carnegiella strigata* (family Gasteropelecidae), both also leave the water, moving along a ballistic aerial path, in response to startle stimuli [19], [20]. The African butterfly fish (4–6 cm body length) inhabit the first few centimeters below the water surface and will go into the air when startled. It is still unclear if the fish are active or passive during the aerial excursion, but jumps are a result of single pectoral fin abduction and not by a tail flip. Jumps sometimes include body rolls [20]. These fish posses a brainstem startle circuitry, including a pair of Mauthner cells but lack the stereotypical lateral startle response. Instead, they perform a vertical startle response that can occur completely within water (2.5–8.4 cm in height) and into the air (2.25–6.6 cm in height) [20]. The phylogenetically unrelated hatchet fishes also have extended pectoral fins, hypertrophied pectoral abductor muscles and jump out of the water when startled, the mechanism produces this modified startle response is achieved through the Mauthner mediated circuitry. [21]. Hatchet fishes can jump either away or towards the stimulus to land behind it in the latter case. Fish have either vertical (up to ∼1.5 body lengths in height and ∼1 body length in distance) or horizontal trajectories (up to ∼1 body length in height and ∼ 4 body lengths in distance, [21]).

A third reason fishes leap into the air is to overcome obstacles in their migration path. The Sockeye salmon, *Oncorhynchus nerka,* and the brook trout, *Salvelinus fontinalis*, negotiate objects that are blocking their path in the stream by leaping over them [22], [23]. Salmon in Alaska are able to jump up to 2.7 body lengths (∼170 cm) at takeoff speeds of approximately 0.5 m/sec [22], [23]. During migration in Colorado, the brook trout will jump as high as 4.7 body lengths (∼60 cm) when small in size (∼15 cm in length), but only 3.0 body lengths when bigger (20 cm long or more) [23]. The heights of these jumps are correlated to the depth of the pool prior to the obstacle; shallower pools constrained the height of the jumps in larger animals. The trout and salmon jumps are both produced in water currents, creating a very specific type of kinetic environment for the generation of leaps.

Lastly, some fish will take advantage of terrestrial habitats [24]. Some teleosts will voluntarily make use of land to evade predators or escape poor conditions. This behavior has been observed for killifishes (Cyprinodontiformes) and several different species have been observed to move across land via a “tail flip” behavior that generates a terrestrial jump. In *Gambusia affinis* (a killifish, Cyprinodontiformes) and *Danio rerio* (a small carp, Cypriniformes; both fishes are about 4 cm in body length) use tail flip-driven terrestrial jumps as a escape, which are kinematically distinct from aquatic escapes [24].

### II. The Trinidadian Guppy

**Figure 1:**
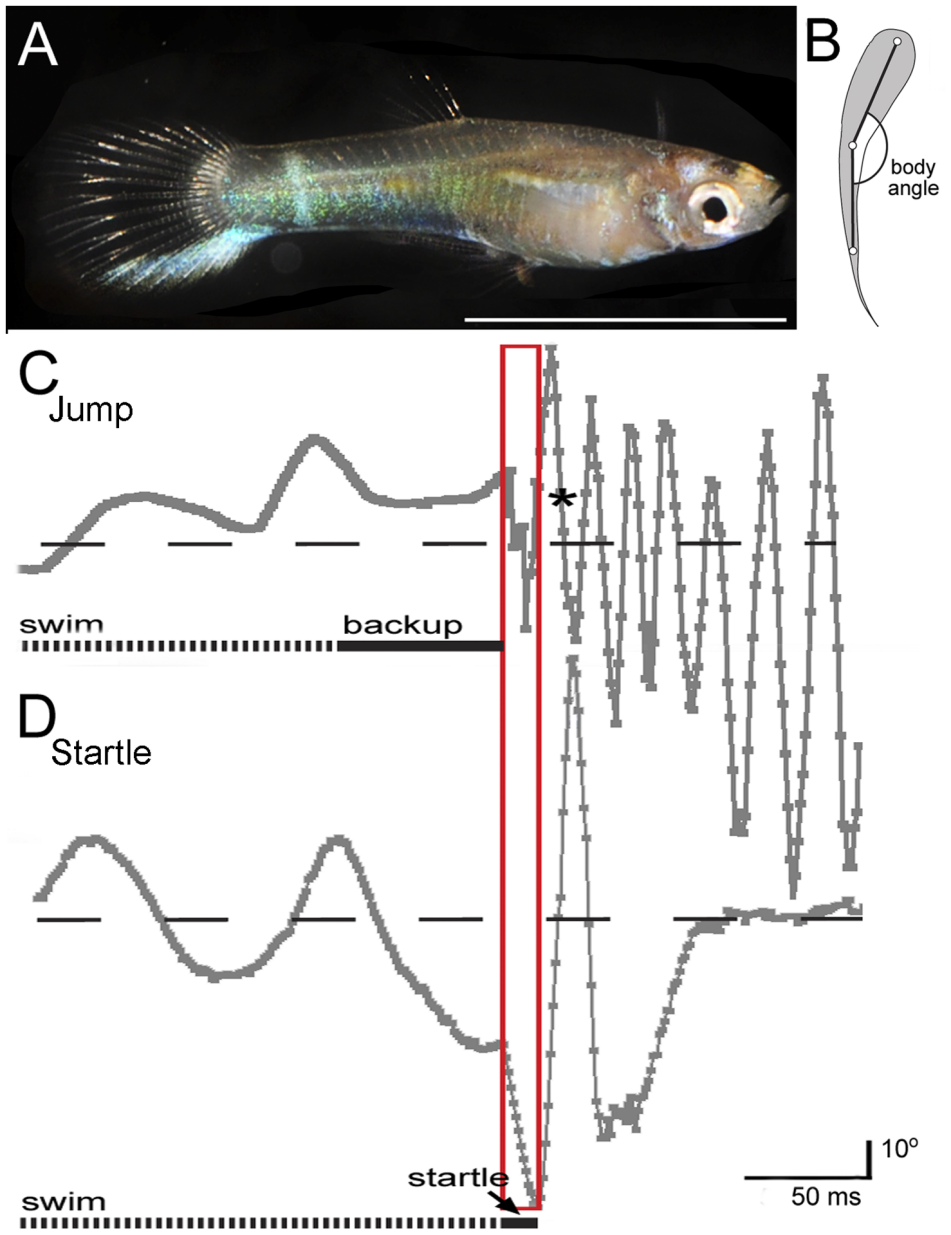
*Poecilia reticulata* and body angle analysis. (*A*) Male Trinidadian guppy, *Poecilia reticulata*. Scale bar = 1 cm. (*B*) Schematic of digitized points and method for measuring body angle. (*C*) Plot of body angle over time during a jump. * Indicates the fastest velocity of the fish, ¾ of body length out of the water. (*D*) Plot of body angle over time during a startle response. Horizontal dashed lines in the centers of the angle diagrams represent straight (180°) body positioning. The red box marks the first stroke of the jump and stage 1 of the C-start.

Guppies (*Poecilia reticulata*; Figure 1A) in Trinidad have rapidly evolved in response to environmental pressures and are a well-established animal model for the study of ecology and evolutionary biology [25]–[28]. This live-bearing fish is common in the northern mountains of Trinidad and is endemic to streams that vary in their ecological characteristics [29]. Crispo et al. (2006) [30] argued that geographical features have had substantial effects on the genetic structure and evolution of this species, with waterfalls substantially reducing gene flow [31]. Fishes from the lower parts of the streams have more allelic diversity than those found upstream and are believed to reflect an older, perhaps original population [32], [33]. Downstream guppies have repeatedly and independently colonized and adapted to upstream environments [34], resulting in parallel, rapid changes in life-history traits, behavior and morphology [26], [28], [35], [36]. This dispersal has been partially constrained by geological features [31], but is strongly driven by high levels of predation in the lowlands [25], [30], [37].

Here, we describe the kinematics of spontaneous jumping behavior of the male guppy from high predation stocks. We take advantage of the well-described ecology and evolutionary history of guppies and suggest possible roles that the jumping behavior might play in their dispersal.

## Methods

All experiments and animal-care activities were approved by the institutional Animal Care and Use Committee of the Marine Biological Laboratory (Woods Hole, MA). Fish were borrowed from Dr. Kim Hoke at the Colorado State University (CSU) and were shipped to the Marine Biological Laboratory to be used in a parallel study.

### Collection and Rearing in the Laboratory

Female guppies were collected from the Guanapo River, a high-predation locality in the Northern Range Mountains of Trinidad [38]. Second-generation family lines, from 20 to 30 wild-caught gravid females, were established by Dr. Cameron Ghalambor in 2008 at CSU to reduce environmental and maternal influences (see details in [39]). Male guppies from this colony were shipped to the Marine Biological Laboratory in the summer of 2011, housed in custom-made tanks with individual flow-through systems on a 12∶12 hour light cycle that were kept in a temperature-controlled chamber. The fish were fed a limited diet twice daily (Tetramin tropical fish flake paste). All fish were mature at the time of the experiment.

### Video Recordings

Fish jumped spontaneously out of their home tank (so that tight grid covers are necessary) and jumped in the experimental tank after a few minutes of adaptation. No stimulation was necessary to trigger jumping at any point. For this study, fish were individually housed for 5 to 20 minutes in a custom-made Plexiglass rectangular arena (10×25×25 cm). All of the sides of the tank except for one were made opaque and we placed a mirror on the clear side so as to be able to film the fish jumping dorsally and laterally simultaneously. Most (7 out of 11) of the fish started spontaneously jumping after a few minutes. Two (2 of 11) fish spontaneously jumped after about an hour and the remaining two (2 of 11) did not jump at all. Each jump was recorded using high-speed videography (X-PRI camera; Del Imaging, Cheshire, CT) at 1000 frames per second and AOS imaging light software (Del Imaging). Twenty jumps performed by five male fish were recorded in this set-up. Recordings from another experiment performed in a different tank, in which all of the sides were opaque (imaged from above) and in which no mirror was present were also used. Twenty-two jumps of four different fish were recorded in this second set-up, in which only the water component was digitized.

Three points were digitized in this study (Figure 1B): 1) head, midpoint between the eyes, 2) midbody, at the widest part of the body in the abdomen and 3) the base of tail before the fin rays. These were used as references for analyses performed using Proanalyst software (Xcitex, Boston, MA). Measurements of each jump were taken between the frame prior to the start of the jumping preparation period and the frame after the return to the water. On three occasions, fish jumped out of frame or out of the tank. In those cases, the last frame in view was the last to be digitized, but we estimated the peak of the jump by examining the deceleration and path. Fish were photographed, measured and weighed after each session (Table 1). Presented values are means ± s.d. unless stated otherwise. The statistical analysis was performed using SigmaPlot software (Systat, San Jose, CA).

**Table 1:** Descriptions of fish.

Fish standard length (cm)	1.85±0.35
Fish weight (gm)	98±10
Height of jump (cm, body length)	6.54±1.92, 3.52±0.96
Maximum velocity in air (cm/sec)	123.57±30.0
Maximum velocity in water (cm/sec)	102.36 cm/sec ±33.75
Angle of attack of jump	77.4° ±24.91
Depth prior to start of jump (cm,body length)	1.86±1.40, 1.04±0.73

**Table 2:** Statistical data from guppy jumps.

Jumping height vs.:		*R* ^2^	*F*	*p*
	Velocity in water	0.7499	41.9787	<0.0001
	Velocity in air	0.6447	25.4037	0.0002
	Depth before jumping	0.8076	62.9712	<0.0001
	Back-up distance	0.6352	12.1901	0.0101

## Results

**Figure 2:**
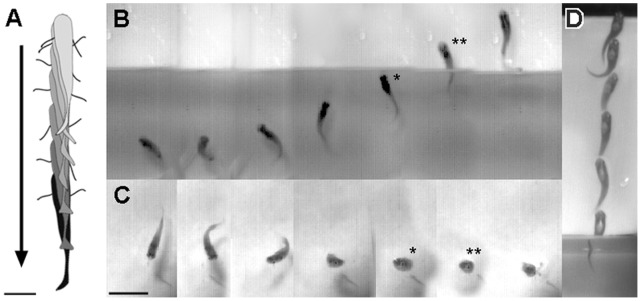
Time-series of guppy jumping behavior. (*A*) Silhouette series of the preparation period of a jump showing backward swimming using fins and without body undulations. Arrow shows the direction of movement and silhouettes are shown at 120-msec intervals. Scale bar = 0.5 cm. (*B–C*) The jumping portion of the behavior viewed from the side (*B*) and from above (*C*). Images are shown at 10 msec intervals. * Indicates the moment of highest speed in the water (128 cm/sec) and ** indicates the highest speed in the air (150 cm/sec). (*D*) Example of the out-of-the-water portion of jump, shown in a different fish that reached height of 6.2 cm. Frames were overlaid at 12 msec intervals. Scale bar = 2 cm.

Guppies spontaneously jumped out of the water without being stimulated by a startle stimulus or being attracted by prey. This behavior occurred among groups of fish in their home tanks (not directly tested here, but observed anecdotally) and when fish were isolated in individual tanks. All fishes were quiescent before jumping and never jumped while exploring the tank or swimming. From a still position, fish reversed using only their pectoral fins and no body undulations were observed (Figure 1C “back-up” and Figure 2A; Videos S1 and S2). Backward swimming was slow (10±4.0 cm/sec) for 3.09±1.72 cm (0.82±0.40 body lengths) and distance was positively correlated with jump height (*r* ^2^ = 0.64, *p* = 0.01). In contrast, there was a negative (−0.19) correlation (*r* ^2^ = 0.81, *p*<0.0001) between depth and jump height, so that, in general, fish located at shallower depths jumped the highest, with one exception (depth of 2.2 cm and a height of 8.2 cm).

**Figure 3:**
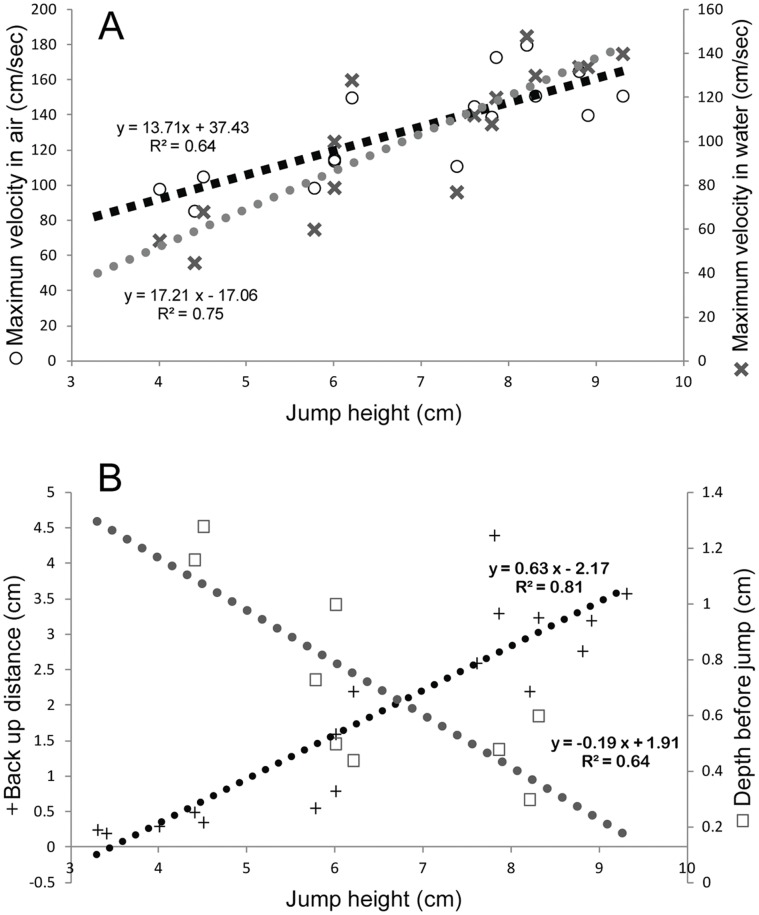
Correlation of jump height with velocity and preparatory-period variables. (*A*) Jump height is positively correlated to greater velocities in water and air. (*B*) Jump height is positively correlated with back-up distances observed during the preparatory period and negatively correlated with depth prior to jumping.

Fish jumped, with adducted pectoral fins (Figure 2B,C,D), from a depth of 1.86±1.40 cm (∼one body length) and reached a height of 6.54±1.92 cm (3.52±0.96 body lengths; Figure 3A, B; Videos S1 and S2). Thrust appeared to be generated by axial body motion. In water, maximum velocities (102.36±33.75 cm/sec) were observed right before or at the moment the fishes’ head broke the water/air interface. The peak velocities in the air were slightly higher (123.57±30.0 cm/sec), presumably because of decreased drag, and were achieved immediately after breaking the interface, at approximate ¾ to 1 body length out of the water. Higher peak velocities yielded higher jumps (Figure 3A). Jump height was positively correlated with the maximum velocity in the air (13.7, *r* ^2^ = 0.64, *p* = 0.0002) and in the water (17.21, *r* ^2^ = 0.75, *p*<0.0001). The average fish attack angle, measured as the angle between the fish’s head and surface of the water, was 77.4°±24.91° and fish with attack angles close to 90° jumped the highest. Typical fast body thrusts initiated the jump sequence, in which the first bend from straight (180°) to maximum curvature (65°±6.6°) took 7.8±0.5 msec and was followed by a faster second bend, with 8.0±1.0 msec from the maximum bend to the next maximum bend.

The jumping cycle started with fast body thrusts, so that the velocity, angle and performance of the first two body wall contractions resembled escape responses. This suggests the possible recruitment of the same neural circuitry responsible for startle behaviors. Jumping behavior fits into a larger category of fast-start behavior, including C-start and S-start escapes and feeding [40]–[42]. Several kinematic similarities were observed between jumping and fast C-start escapes. Similar to many other species, guppies perform a typical C-start startle response with a stage 1 C-bend and stage 2 reverse propulsive stroke (Figure 1D; [43], [44]). Jumping behavior started with a strong unilateral body bend, similar to stage 1, which was followed by an oppositely directed propulsive bend, similar to stage 2 (Figure 2C second and third frames, top view). The timing between body bends was comparable in the two behaviors (Figure 1C, D), but further detailed kinematic studies are needed to compare the absolute and relative durations of the different stages and the maximum curvatures of guppy C-starts. Following the first two body wall contractions, the jumping behavior continues with a series of high frequency axial bends (Figure 1C). These movements are similar to burst swimming, which follows a C-start (Figure 1D; [20], [45]). In jumping, propulsive movements may allow the animal to reach the peak velocities observed as the fish break the water/air interface. The burst speed, achieved with a C-start in animals of the same species and length, is about 70 to 80 cm/sec (extrapolated from [45] and this study). In comparison, the fastest speed we recorded in water was 100.16±33.9 cm/sec, and in the air, we recorded at top speed of 123±30.0 cm/sec.

## Discussion

The Trinidadian Guppy, *Poecilia reticulata* is notable for its fast evolution and habitat. Guppies are common in the northern mountains of Trinidad and are endemic to streams that vary in their ecological characteristics [29]. Fishes from the lower parts of streams share habitats with predators and have repeatedly, independently colonized and adapted to upstream environments that contain no predators [34]. This has led to parallel, rapid changes in life-history traits, behavior and morphology [26], [28], [35], [36]. We measured the characteristics of spontaneous jumping in Guppies that were bred in the laboratory from high predation sites. These fish will spontaneously jump out of the water without being stimulated by a startle stimulus, or areal prey items and are not under seasonal migration pressure. Here, we quantified this behavior and demonstrated that it includes a preparatory phase of slow backward swimming, followed by fast forward swimming and an aerial phase. No descriptions of areal jumping in fishes up until now show this preparatory backward swimming phase. We also demonstrated that the first two body bends of the jump share kinematic similarities with a C-start behavior. The preparatory backward swimming prior to jumping does not exclude the possibility that this behavior may be C-start behavior, as “anti-predator posture” movements have been observed and related to C-start responses in other species [46]. Further examination of startle kinematics and jumping physiology is needed before any conclusions can be made about a shared neural substrate. These similarities do not necessarily imply the involvement of the Mauthner cells, but may suggest the involvement of elements of the C-start response circuitry. Given the high-speed nature of jumping kinematics, the sudden onset of the jumping behavior and the high cost of developing and maintaining the neural circuitry needed to drive such behavior, it is reasonable to consider the possibility that some of the same circuitry elements may be used in both of these jumping and C-start.

It is possible that guppies also jump out of the water as a form of startle response, but it is unlikely that jumping is involved in seasonal migration [47], since guppies are not known to change territories seasonally. There is also no evidence to date that guppies feed on arboreal food items like the archer fish or the Arowana. Previously, Wöhl and Schuster [42] (2007) argued that the predictive start of a hunting archer fish is driven by a modification of the C-start reticulospinal startle circuitry. One report has suggested that, in the gulf sturgeon *Acipenser oxyrinchus*, jumping may be involved in acoustic communication [48], but this hypothesis is so far unique and has yet to be developed in guppy.

Because guppy jumping events start slowly with a preparatory phase, and occur without external stimulation, we hypothesize that a jumping behavior is deliberate and has been selected as a strategy for dispersal. Dispersal is advantageous for avoiding competition among kin [49]–[51] and for preventing inbreeding [52]–[54] and also plays crucial roles in population dynamics, species persistence, maintenance of genetic variability, preservation of biodiversity and speciation (see [55]–[57] for reviews). The hypothesis that jumping is adaptive for dispersal could be further tested through comparative studies of upstream and downstream populations. If local habitat adaptation becomes dominant, then it can be predicted that secondary populations (upstream with low predation) are not under the same dispersal pressures as the original (downstream, high predation) populations and that the original high-performance jumper founder population will eventually lead to a decrease in jumping probability and performance. Such changes in dispersal phenotype after colonization of a new habitat have been noted in the literature. Charles Darwin [58] for example, reported that many bird species endemic to islands have lost their ability to fly after colonization. Similarly, insect species that have colonized islands have become flightless [59]–[62]. Future studies should include comparisons between populations from locations with high and low levels of predation, as well as comparisons of the kinematics of males and females. Male guppies have been shown to move from their pool of origin more frequently than females and the probability of emigration is significantly biased toward upstream movement [63]. Therefore, it is possible that jumping is more prominent among males from high predation sites than among other groups.

## Supporting Information

Video S1 **High speed video of guppy jumping viewed from the top.** Notice the preparation phase.
(WMV)

Video S2 **High speed video of guppy jumping viewed on a split screen.**
(WMV)
